# Combining humic acid with phosphate fertilizer affects humic acid structure and its stimulating efficacy on the growth and nutrient uptake of maize seedlings

**DOI:** 10.1038/s41598-020-74349-6

**Published:** 2020-10-15

**Authors:** Jianyuan Jing, Shuiqin Zhang, Liang Yuan, Yanting Li, Zhian Lin, Qizhong Xiong, Bingqiang Zhao

**Affiliations:** 1 Key Laboratory of Plant Nutrition and Fertilizer, Ministry of Agriculture and Rural Affairs / Institute of Agricultural Resources and Regional Planning, Chinese Academy of Agricultural Sciences, Beijing, 100081 China; 2 Anhui Province Key Laboratory of Farmland Ecological Conservation and Pollution Prevention, School of Resources and Environment, Anhui Agricultural University, Hefei, 230036 China

**Keywords:** Plant sciences, Materials science

## Abstract

This paper analyzed the compositional and structural changes of humic acid (HA) after combined with phosphate fertilizer (PHA), and investigated its effects on the growth of maize seedlings with four humic acid concentrations. The results showed that the atomic ratios of O/C and (O + N)/N of PHA were significantly lower than those of HA, which indicated that PHA had poor hydrophilicity compared with HA. The spectra of FTIR and NMR results suggested that the relative content of carboxyl group in PHA was higher than that in HA. X-ray photoelectron spectroscopy technology showed that the relative amount of C–C in PHA was lower than that in HA, while C–H was the opposite. The above changes were attributed to the crack of HA structure during the preparation of humic acid enhanced phosphate fertilizer, which was verified by the results from the determination of gel permeation chromatography that there were more low molecular weight components in PHA than that in HA. However, compared with HA, PHA showed a worse effect in promoting growth and the uptake of nitrogen, phosphorus and potassium by maize seedlings. This worse effect might be attributed to the poor hydrophilicity and unsuitable addition amount of PHA.

## Introduction

Humic acid is an organic compound derived from plant and animal residues and microbial cells with long-term physical, chemical, and biological processes, and it is also a natural material that improves the efficiency of phosphate fertilizers^1^. Many studies have shown that humic acid co-applied with monocalcium phosphate can increase the movement and availability of P in soil^2,3^. Furthermore, humic acid can enhance the efficiency of phosphate fertilizers by promoting H^+^ release in the rhizosphere and increasing phosphate uptake by plants^4–6^. However, in the above studies, humic acid is always applied to the soil in large amounts.

In recent years, conventional phosphate fertilizers combined with trace amounts of plant-based biostimulants have become an emerging way to improve the efficiency of phosphate fertilizers, and the manufactured fertilizer is called value-added phosphate fertilizer^7,8^. It shows a distinct advantage based on its large output and low cost. Humic acid is often used in the process of making value-added phosphate fertilizers, and the corresponding product is called humic acid enhanced phosphate fertilizer (HAP), the yield of which reached 500,000 tons in China in 2019.

Field studies have reported that HAP showed a better performance in crop yield and apparent phosphate utilization than common phosphate fertilizers^7,9,10^. One possibility is that the application of HAP reduced the soil pH, increased the available phosphorus content in the soil, and promoted the uptake of phosphorus by plants^8,10^. At the same time, it may be closely related to the formation of bioavailable phosphate nanoparticles stabilized by the etching of phosphate crystals^3^. Humic acid could convert insoluble phosphorus into dissolved phosphorus due to the ion exchange with anions of humic acid and remineralization into more soluble phosphorus^11,12^. In addition, humic acid was also used as phosphorus recycled material and then to stimulate plant growth^13,14^. However, researchers have paid almost all attention to the effect of HAP on the availability and utilization of phosphorus fertilizers, and they have ignored the possibility that the combination with phosphate fertilizers might change the structure and function of humic acid.

During the production process of chemical phosphate fertilizers, the exothermic neutralization reaction of phosphoric acid and alkaline substances occurs, and a high temperature is generated^15,16^. The changed temperature may affect the structure of humic acid. When the temperature of the reaction system reaches 290 °C, the elemental content and atomic ratio of humic acid will change^17^. Zhang et al.^18^ reported that high temperatures (> 400 °C) will also cause a serious loss of humic acid by weight, especially for aliphatic compounds. Zhou et al.^19^ also reported that the temperature of the reaction system is the most important factor affecting the properties of humic acids. However, the effect of the high temperature generated during the production process of HAP on the structure of humic acid has not been reported.

The structural variation of humic acid will also change its biological activity. Compared with raw humic acid, oxidized humic acid with a high yield of small molecular weight, low aromaticity, and low hydrophobicity index can significantly improve the root biomass and activity of maize^20^. By comparing four humic acids from different sources, Jindo et al.^21^ found that humic acid with more carboxylic groups and a high hydrophobicity can promote the growth of the maize root system. García et al.^22^ also reported that humic acid with more labile and functionalized groups is responsible for root emission, while that with more recalcitrant and less functionalized groups is related to root growth. Furthermore, the effect of humic acid on the nutrient uptake of plants is also related to its structure. Albuzio et al.^23^ reported that fractionated humic acids will significantly reduce the nitrate uptake of barley, while the content and type of functional groups of humic acid will be changed after fractionating^24^.

Therefore, we extract humic acid from HAP (PHA) by adjusting the pH of the HAP solution. The objective was to study the changes in the structure of humic acid after combining with phosphate fertilizer and the effects on the growth and nutrient uptake of maize seedlings. The structures of raw humic acid (HA) and PHA were determined by various characterization techniques, and the effects of HA and PHA on the growth and nutrient uptake of maize seedlings were investigated via hydroponic experiments with four concentrations. This research could explain the synergistic mechanism of HAP.

## Results and discussion

### Characterization of HA and PHA

The elemental compositions of HA and PHA are shown in Table 1. HA and PHA exhibited a significant variation in the contents of oxygen, nitrogen, and ash and in the atomic ratios of O/C, N/C and (O + N)/N. Carbon was the main component, accounting for 59.48% and 59.36% of the HA and PHA, respectively, which was consistent with the results of Zhang et al.^24^. Compared with HA, the ash contents of PHA significantly decreased by 30.88% (*P* < 0.05), while the nitrogen content significantly increased by 124.41% (*P* < 0.05). The decrease of the ash content in PHA indicated the loss of inorganic components, which might be attributed to the removal of soluble metal ions during the PHA extraction process. There were no nitrogenous substances involved in the production of HAP, but the nitrogen content showed an obvious increase, which might be related to the change of the other components of the PHA, such as the decrease of ash contents.

**Table 1:** Elemental composition and atomic ratio of the HA and PHA.

Sample	Elemental composition (%)					Ash content (%)	Atomic ratios			
	C	H	O	N	S		H/C	O/C	N/C	(O + N)/N
HA	59.48^a^	2.74^a^	31.08^a^	2.54^b^	0.75^a^	3.40^a^	0.55^a^	0.39^a^	0.04^b^	11.86^a^
PHA	59.36^a^	2.76^a^	29.16^b^	5.70^a^	0.67^a^	2.35^b^	0.56^a^	0.37^b^	0.08^a^	5.59^b^

*HA* humic acids, *PHA* humic acids extracted from humic acid enhanced phosphate fertilizer.

Means with no letter in common are significantly different (P < 0.05), as indicated by the least significant difference (LSD) test (n = 3).

In addition, the hydrophobicity of humic acid had a negative correlation with the relative atomic number of O^25^. Our results showed that atomic ratios of O/C and (O + N)/N of PHA were significantly lower than those of HA, which indicated that PHA had poor hydrophilicity compared with HA.

**Figure 1:**
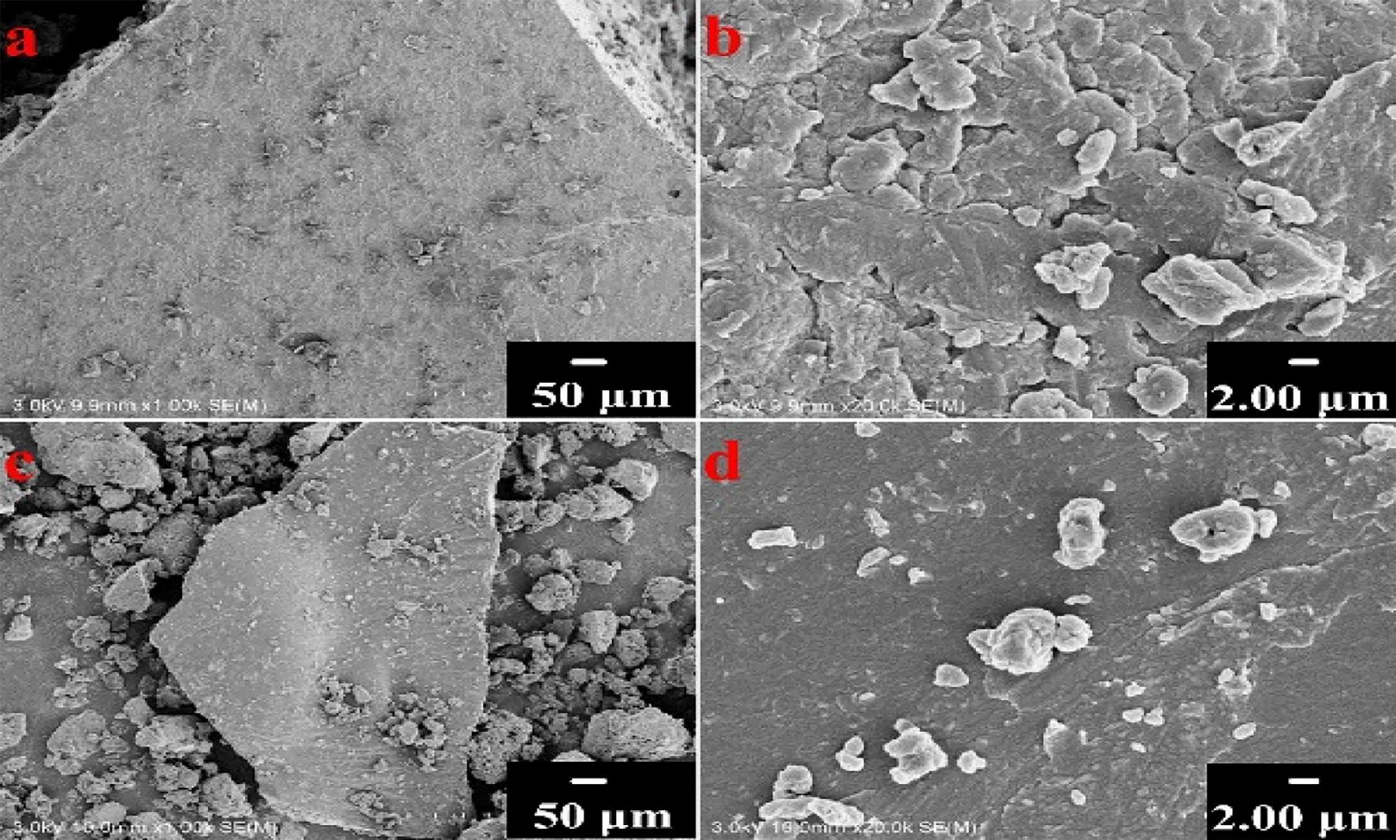
SEM images of HA and PHA. **a**,**b** for HA with magnified 1000 and 20,000 times, respectively; **c**,**d** for PHA with magnified 1000 and 20,000 times, respectively.

The surface morphologies of HA and PHA are shown in Fig. 1a−d. There was no significant difference between PHA and HA, and both of them exhibited a loose and abundant structure with nonhomogeneous pores. This outcome indicated that after combination with phosphate fertilizer, the amorphous structure of humic acid was well-maintained^17^.

**Figure 2:**
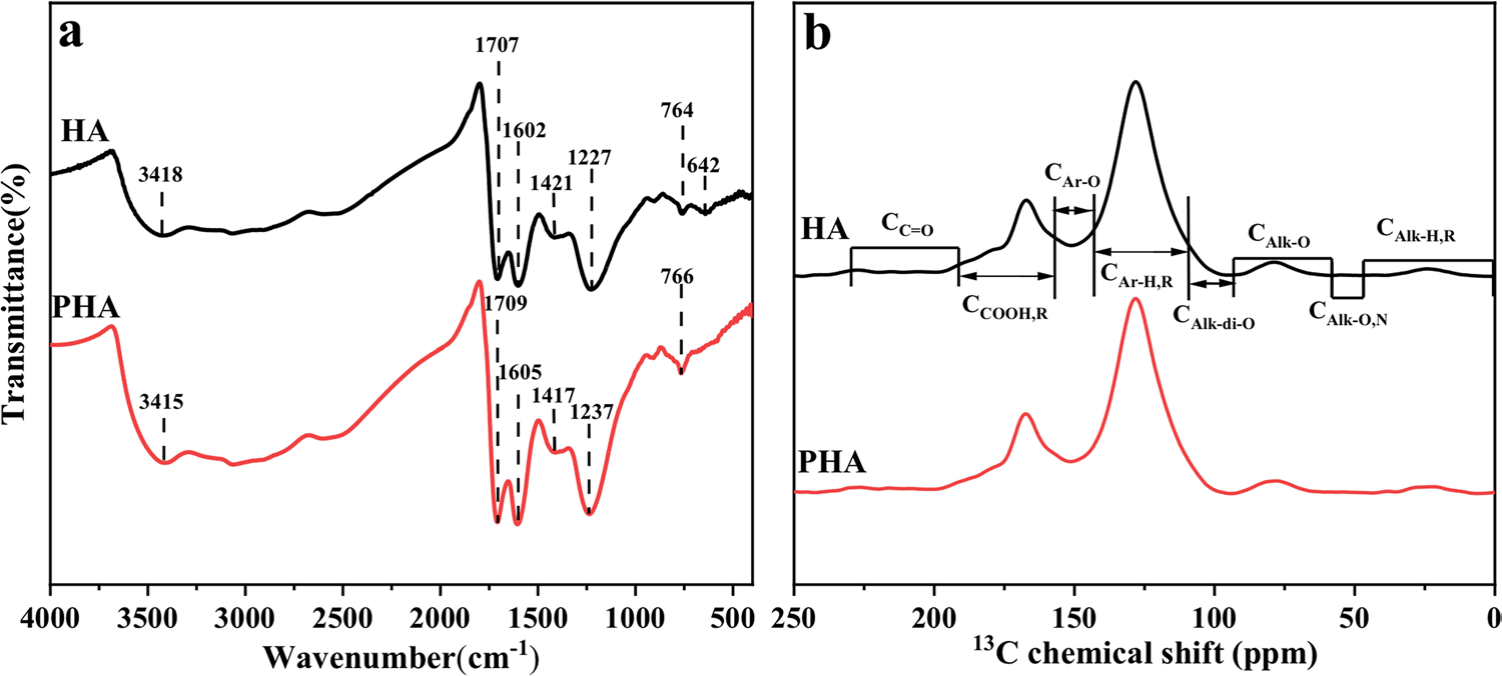
FTIR spectra (**a**) and ^13^C NMR spectra (**b**) of HA and PHA.

The FTIR spectra of HA and PHA are shown in Fig. 2a, and the relative absorption intensity of the main absorption peaks is listed in Table S1 (see Supplementary Table S1 online). PHA showed a similar FTIR spectrum to HA. The peaks at 3418 and 3415 cm^−1^ were attributed to –OH stretching in alcohols and phenols. The stretching vibration of carboxyl group C=O was found at 1707 and 1709 cm^−1^ and the C–O–H in-plane bending vibration of carboxylic acid at 1421 and 1417 cm^−1^^17,19^. According to the integration in Table S1 (see Supplementary Table S1 online), the vibration intensity of the above functional groups in HA was weaker than that in PHA, which indicated that the relative carboxyl content of PHA was higher than that of HA. In addition, compared with HA, the vibration intensity of PHA at 900−600 cm^−1^ decreased (see Supplementary Table S1 online), which indicated that the ash in the PHA was reduced, similar with the result of the elemental composition (Table 1). These FTIR spectra results suggested that during the preparation of HAP, the main functional groups of humic acid were not changed, while the relative content of carboxyl group increased.

HA and PHA had similar NMR spectra (Fig. 2b), and both of them had a high representation of aromatic C (58.79% for HA and 58.16% for PHA; Table 2), followed by carboxyl carbon (20.61% for HA and 20.86% for PHA). The relative content of carboxyl carbon in PHA was 1.2% higher than that in HA, which was consistent with the FTIR results in Table S1 (see Supplementary Table S1 online) but inconsistent with the results of Kolokassidou et al.^26^, who reported that decarboxylation occurred at temperatures over 130 °C. This inconsistency occurred because in this study, the high temperatures caused by the reaction between phosphoric acid and potassium hydroxide was transient, while a slow increase in the temperature of the reaction system was needed when the decarboxylation reaction happened. In addition, the ratio of carboxyl group (the proportion of acidic functional groups) showed no significant difference between HA and PHA, but that of PHA7 was higher than that of HA7 (see Supplementary Table S2 online). This outcome suggested that PHA contained more carboxyl groups than HA, while parts of the groups in PHA were present in the form of carboxylate. The oxygen-containing and nitrogen-containing alkyl carbons in humic acid were hydrophilic carbons^25^. We found that relative abundance of methoxy and N-alkyl carbons, O-alkyl carbons, and dioxide-alkyl carbon in HA was 6.45 (Table 2), while those carbon types of PHA was 6.31. This further indicated that PHA was less hydrophilic than HA.

**Table 2:** Relative abundance of different carbon types (%) as determined by the ^13^C RMN by CP/TOSS techniques for HA and PHA.

C Type ppm	C_Alk-H,R_ 0−45	C_Alk-O,N_ 45−60	C_Alk-O_ 60−91	C_Alk-di-O_ 91−110	C_Ar-H,R_ 110−142	C_Ar-O_ 142−156	C_COO-H,R_ 156−186	C_C=O_ 186−230
HA	1.95	0.19	3.40	2.86	58.79	8.05	20.61	4.16
PHA	2.06	0.27	3.26	2.78	58.16	8.16	20.86	4.45

**Figure 3:**
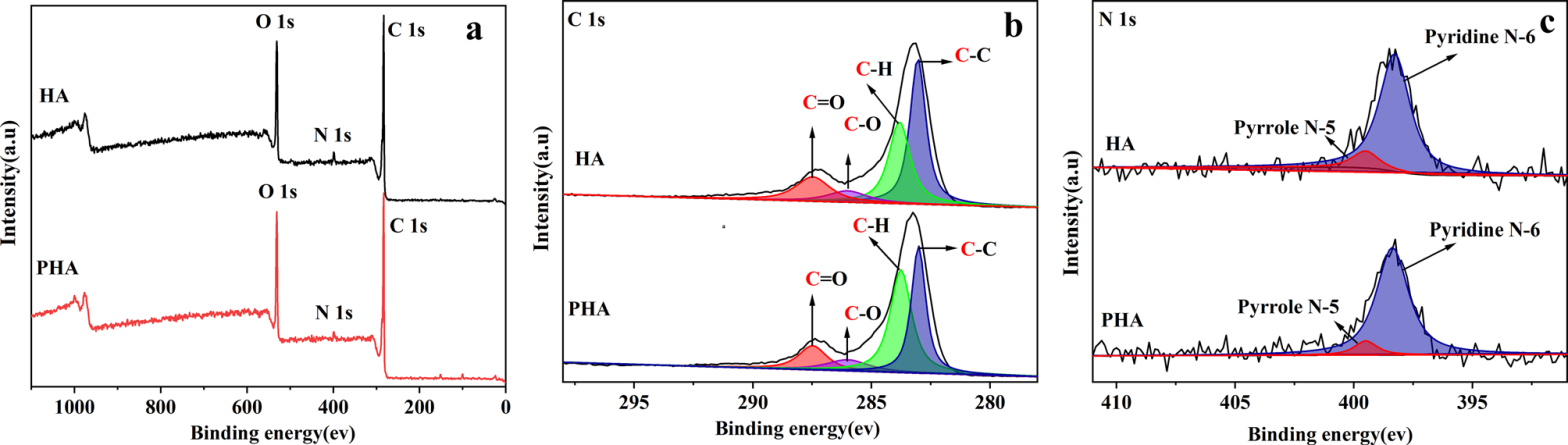
XPS spectra (**a**), C1s (**b**), and N1s (**c**) spectra of HA and PHA. Top: HA, bottom: PHA.

XPS survey spectra confirmed the presence of C, N and O in both HA and PHA (Fig. 3a). Figure 3b shows the high-resolution C1s spectra of HA and PHA. The binding energy peaks centered at 283.04, 283.82, 286.00, and 287.50 eV for HA and at 283.01, 283.76, 286.00, and 287.48 eV for PHA are assignable to C–C, C–H, C–O, and C=O, respectively (see Supplementary Table S3 online)^27–30^. Compared with HA, the relative amount of C–C in PHA decreased by 18.5%, while C–H increased by 29.6%, which indicated that the relative proportion of protonated C was higher than that of HA. Therefore, the long chains of humic acids were broken in the process of manufacturing HAP.

The peak in the N1s spectra of HA and PHA was assigned to Pyridine N-6 and Pyrrole N-5 (Fig. 3c)^31–33^. The peak at 398.27 eV with 88.8% area of HA N1s spectra indicated the presence of pyridine N-6, and the peak at 399.50 eV with 11.2% area was attributed to pyrrole N-5. Compared with HA, PHA had more Pyridine N-6 (398.37 eV, 91.6%) and less Pyrrole N-5 (399.49 eV, 8.4%) (see Supplementary Table S4 online). This result was consistent with the result of the severely pyrolyzed chars (> 600 °C)^34^.

O1s of X-ray photoelectron spectroscopy confirmed that PHA had more C=O as carboxyl (see Supplementary Fig. S1 and Table S5 online), in accordance with the FTIR (see Supplementary Table S1 online) and ^13^C NMR results (Fig. 2b, Table 2). In addition, the XPS spectra (Fig. 3a) and ^31^P NMR analysis (see Supplementary Fig. S2 online) confirmed the absence of P in PHA, which indicated that the humic acid and phosphate in HAP could be separated by adjusting the pH of the HAP solution.

**Figure 4:**
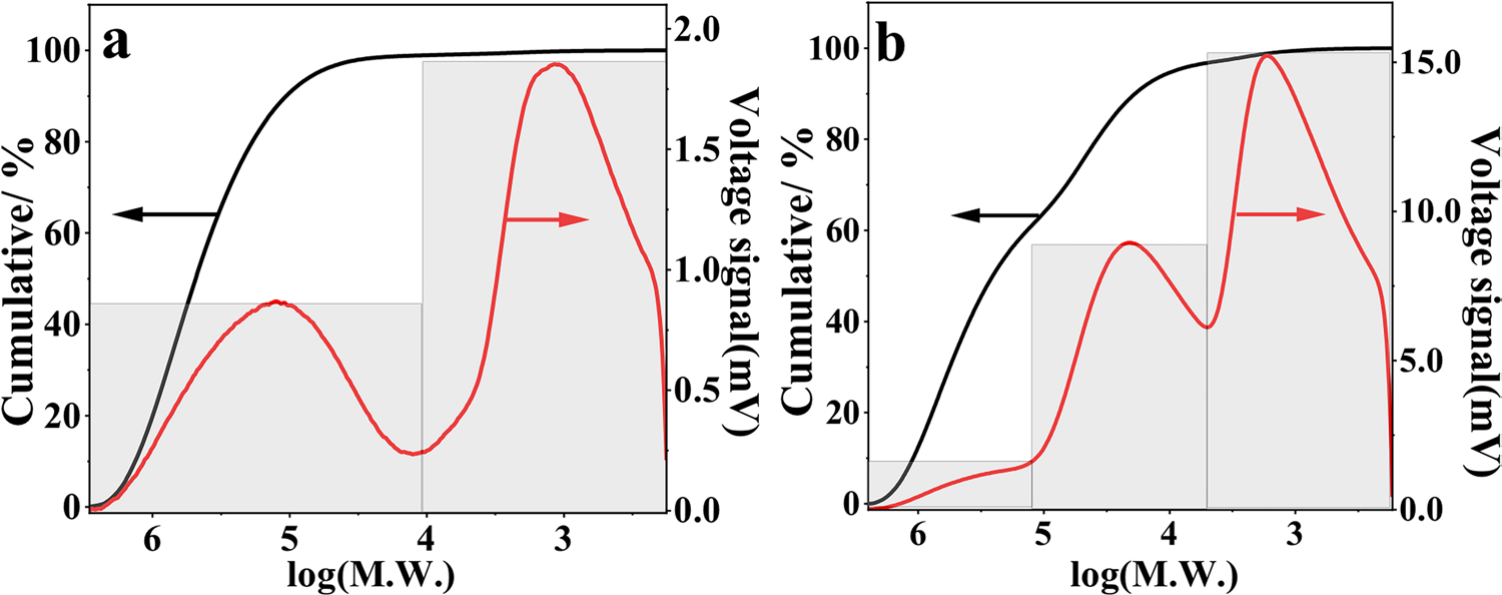
Molecular weight distribution curve of HA (**a**) and PHA (**b**).

Gel permeation chromatography was conducted to determine the molecular weight distribution of HA and PHA. According to the molecular weight distribution curve (Fig. 4a,b, Table 3), PHA had more components with small molecules than HA. HA had two groups, while PHA had three groups of different molecular weights. The two main molecular weight distribution areas of HA were 2.25 × 10^2^−8.25 × 10^3^ Da and 1.37 × 10^4^−2.88 × 10^6^ Da, accounting for 51.9% and 48.1% of all detected molecular weights, respectively. However, PHA had more small molecules, and the molecular weights ranged from 1.70 × 10^2^−4.92 × 10^3^ Da accounting for 61.4% of all detected molecular weights. Therefore, during the preparation of humic acid enhanced phosphate fertilizer, some components of humic acid were decomposed to generate more small molecules.

**Table 3:** Molecular weight distribution of HA and PHA.

Humic acids type	Main peaks (Da)	Interval M_W_ (Da)	Area (%)	Mw/Mn
HA	1.47 × 10^3^	2.25 × 10^2^ − 8.25 × 10^3^	51.9	1.52
	1.26 × 10^5^	1.37 × 10^4^ − 2.88 × 10^6^	48.1	2.60
PHA	1.67 × 10^3^	1.70 × 10^2^ − 4.92 × 10^3^	61.4	2.00
	2.09 × 10^4^	5.03 × 10^3^ − 1.45 × 10^5^	35.1	1.88
	1.51 × 10^5^	1.51 × 10^5^ − 2.89 × 10^6^	3.5	1.45

### Growth of the maize seedlings

**Figure 5:**
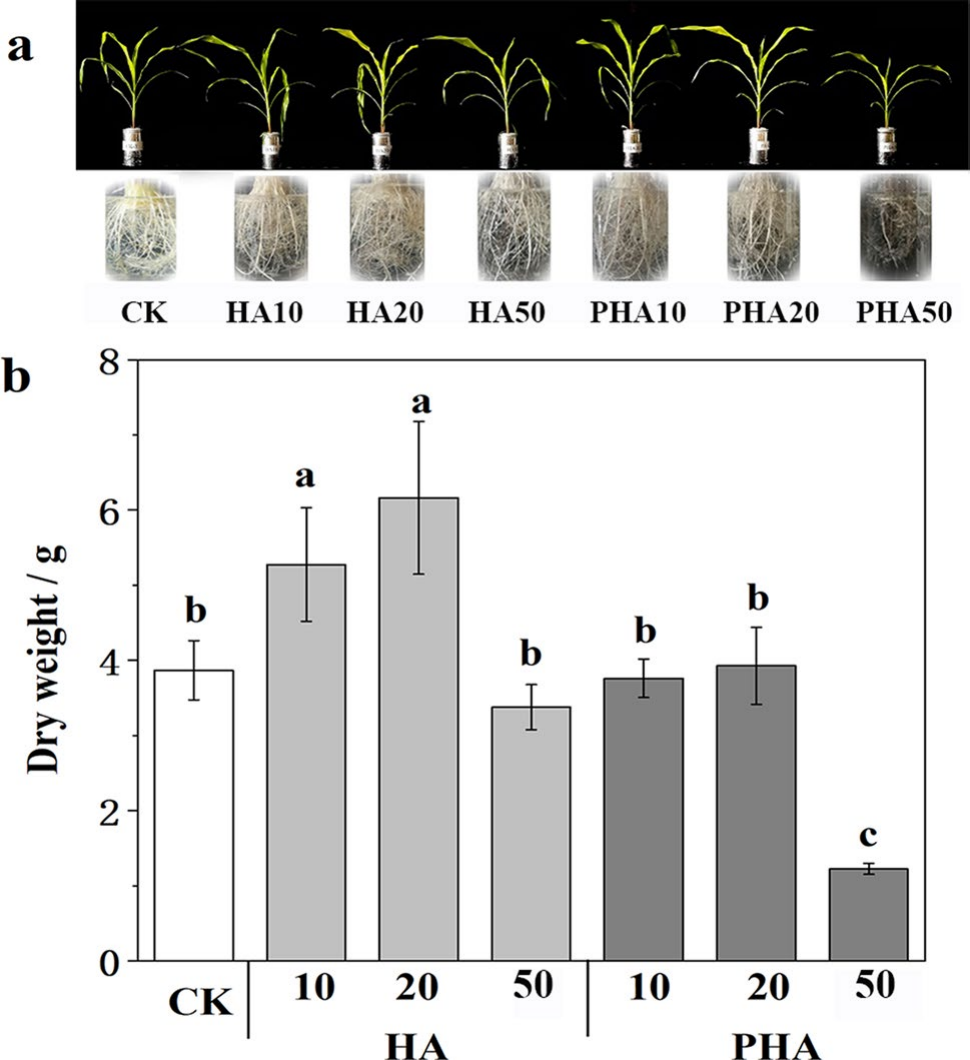
Growth status of ground and root of maize under different amounts of HA and PHA (**a**) and dry weight of maize with different amounts of HA and PHA (**b**). *CK* Hoagland's nutrient solution without humic acids, *HA10, HA20, and HA50* Hoagland's nutrient solution with humic acids of 10, 20, and 50 mg C/L, *PHA10, PHA20, and PHA50* Hoagland's nutrient solution with PHA of 10, 20, and 50 mg C/L. Bars represent mean ± SD (n = 5). Means with no letter in common are significantly different (*P* < 0.05), as indicated by the Tukey’s HSD test.

HA stimulated maize seedling growth at low concentrations but inhibited growth at high concentrations, in accordance with the results from Chen et al*.*^35^. However, the significant promoting effect has not been investigated when PHA is added at low concentrations, and PHA showed inhibition effects at high concentrations (Fig. 5b). Compared with CK, HA10 and HA20 (10 and 20 mg C/L) significantly increased the dry weight of plants by 36.2% and 59.2% (*P* < 0.05), respectively. There was no significant difference between the treatment with HA50 and CK (*P* > 0.05). However, PHA10 and PHA20 showed a similar stimulation with CK, and the dry weight with PHA50 was significantly lower than that with CK (*P* < 0.05). Under the same concentration, the dry weight under HA treatments was significantly higher than that of the PHA treatment (*P* < 0.05), so the growth promotion effect of PHA was inhibited compared with that of HA. This result indicated that the structural difference between HA and PHA affected their stimulating effect on plant growth, and combination with phosphate fertilizer may weakened the biological activity of humic acid.

### Nutrients uptake of the maize seedlings

The N, P, and K uptake of maize increased and then decreased as the amount of added HA increased (Table 4). When the amount of added HA was 20 mg C/L, the uptake of N, P, and K in the maize was significantly higher than that of CK (*P* < 0.05). The effect of PHA in different amounts on plant nutrient absorption varied (Table 4). When the amount of added PHA was 10 and 20 mg C/L, the N uptake showed no significant difference with CK (*P* > 0.05), but it decreased for large amounts of added PHA. PHA and HA had similar effects on the P and K uptake: absorption was promoted at low concentrations and inhibited at high concentrations. However, for the same amount of added carbon, the P or K uptake of HA was higher than that of PHA. Therefore, PHA also had the function of promoting the absorption of P and K by plants, but its function was inhibited compared with that of HA, and the inhibitory effect became more obvious as the content of PHA increased.

**Table 4:** Nitrogen (N), phosphorus (P) and potassium (K) uptake of the maize under different amounts of HA and PHA.

Treatment	N uptake (mg N plant^−1^)	P uptake (mg P plant^−1^)	K uptake (mg K plant^−1^)
CK	130.43 ± 5.23^c^	34.75 ± 3.19^c^	221.09 ± 4.18^bc^
HA10	179.27 ± 9.64^b^	47.90 ± 0.91^b^	293.14 ± 3.71^a^
HA20	206.78 ± 16.79^a^	61.78 ± 2.73^a^	298.68 ± 44.49^a^
HA50	123.28 ± 7.54^c^	36.74 ± 3.41^c^	177.78 ± 5.17^c^
PHA10	121.93 ± 1.86^c^	45.59 ± 1.93^b^	271.02 ± 4.48^ab^
PHA20	121.78 ± 11.02^c^	44.17 ± 1.99^b^	240.45 ± 13.95^b^
PHA50	45.28 ± 1.31^d^	13.08 ± 0.96^d^	82.45 ± 4.41^d^

*CK* Hoagland's nutrient solution without humic acids, *HA10, HA20, and HA50* Hoagland's nutrient solution with humic acids of 10, 20, and 50 mg C/L, *PHA10, PHA20 and PHA50* Hoagland's nutrient solution with PHA of 10, 20, and 50 mg C/L. Values are mean ± SD (n = 5), means with no letter in common are significantly different (*P* < 0.05), as indicated by the Tukey’s HSD test.

Many studies have shown that the growth promotion effect of humic acids with small molecules was better than that with large molecules, and the better performance should also happen for humic acid with more carboxyl group^36,37^. However, our research is likely to show an opposite result. This opposite result might be explained by the application amount of PHA. García et al.^22^ reported that humic acid with more reactive functional groups might promote root stimulation at lower concentrations, while that with recalcitrant structures requires higher concentrations to promote a similar stimulus. In this study, the best dosage of PHA for plant growth and nutrient uptake might even be lower than 10 mg C/L. Zhou et al.^38,39^ also reported that humic acid with a small molecular weight and a medium concentration (10 mg C/L) had the most promoting effect on maize plant growth, and when the amount of added humic acid is above 15 mg C/L, maize growth will be significantly inhibited. In addition, with further observation, we found that there were more humic acid materials attached to the root surface under the PHA treatments compared to the HA with the same carbon additions (Fig. 5a), which might be attributed to the hydrophilicity decrease of organics when the high temperature occurred during the preparation of phosphorus fertilizer^27^. The attachments blocked the absorption channel of mineral nutrients in the root system, leading to the decrease of the nutrient uptake.

## Conclusion

The high temperature, generated during the preparation process of humic acid enhanced phosphate fertilizer (HAP), cracked the structure of humic acid, and increased the relative amount of carboxyl groups and low molecular components. The hydrophobicity of PHA was also increased. Raw humic acid stimulated the maize seedlings growth and uptake of nitrogen, phosphorus and potassium. However, after combined with phosphate fertilizer, PHA showed a worse effect. This changed function might be attributed to the decreased hydrophilicity and unsuitable addition amount of PHA.

## Methods

### Preparation of materials

#### Extraction of humic acid (HA) from weathered coal

Humic acid (HA) was extracted from weathered coal (45°23′ N, 119°15′ E; Huolinhe, Tongliao, Inner Mongolia Autonomous Region, Northeast China) by a modified alkali extraction method, as described by Zhang et al.^24,40^.

#### Manufacture of humic acid enhanced phosphate fertilizer (HAP)

**Figure 6:**
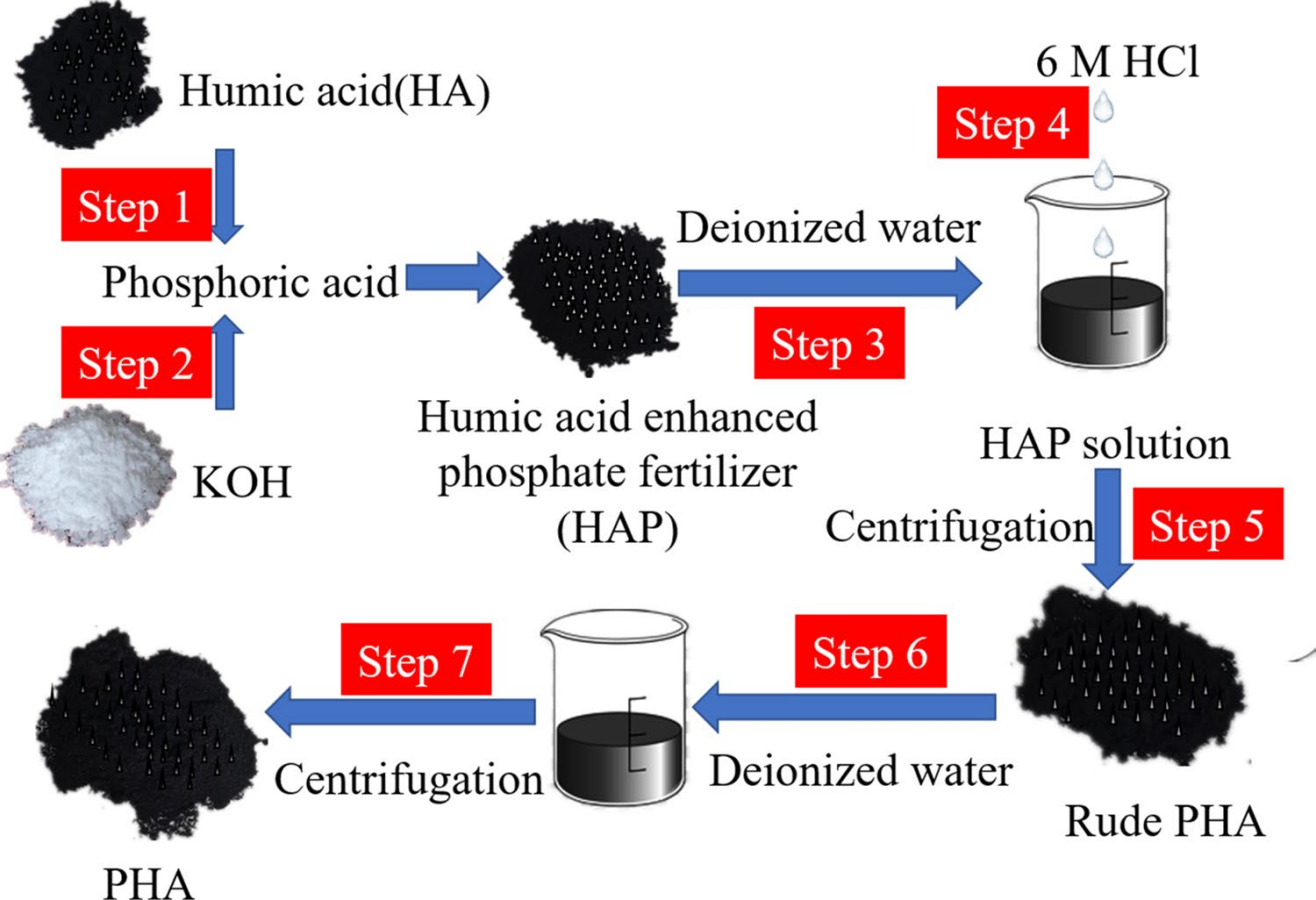
Preparation of humic acid extracted from humic acid enhanced phosphate fertilizer (PHA).

Humic acid enhanced phosphate fertilizer was prepared by simulating the production process of phosphate fertilizer in the laboratory, as shown in the flow chart in Fig. 6. A total of 42.64 parts by weight of potassium hydroxide (GR) was added into the mixture prepared by thoroughly mixing 20 parts by weight of HA with 37.36 parts by weight of phosphoric acid (GR). After continuous stirring, humic acid enhanced phosphate fertilizer was obtained.

#### Preparation of humic acid from HAP

HAP was dissolved in deionized water with a solid–liquid ratio of 1:10, and the solution pH was adjusted to 1.0 by 6 M HCl. After standing for 12 h, the solution was centrifuged to remove the soluble PO_4_^3−^, K^+^, and other ions, and the insoluble parts were collected as crude humic acid in HAP. Then, the crude humic acid in HAP was washed with deionized water at a solid–liquid ratio of 1:10 three times and oven-dried at 50 °C to obtain the final humic acid in HAP (PHA). A schematic diagram of the preparation of humic acid from HAP is shown in Fig. 6.

### Characterization of HA and PHA

#### Elemental analyses

The C, N, H, and S contents of HA and PHA were determined using an element analyzer (Vario Micro Cube, Elementar Analysensysteme GmbH, Germany), and the ash content was determined by burning in a muffle furnace. The oxygen content was calculated by subtracting.

#### Carboxylic groups and phenolic hydroxyl groups

The contents of the carboxylic groups and phenolic hydroxyl groups of HA and PHA were determined by the methods described by Klavins et al. and Zhang et al.^24,41^. To reduce the difference in the carboxylate contents in HA and PHA on the comparison of the carboxylic acid content, the pH of the HA and PHA was adjusted to 7.0, and then their acidic functional groups were compared. The adjustment of the pH was conducted according to the following process: 10 g of HA or PHA was mixed into 100 mL of deionized water, and then the pH of the HA and PHA solution was adjusted to 7.0 with 5 M NaOH. After centrifugation and freeze drying, the processed samples were obtained and named as HA7 and PHA7, respectively.

#### Fourier transform infrared spectroscopy

The atom group information of the HA and PHA was determined by a Fourier transform infrared spectrophotometer (Nicolet iS10, Thermo Nicolet Corporation, America). Infrared spectra (IR) were recorded in the 4000−400 cm^−1^ region using the KBr pellet method^24^. The baseline correction and data smoothing correction of the spectra were conducted with the OMINC 8.2 software. The main peak area was integrated to calculate the relative absorption intensity of each functional group using the Origin 9.0 software.

#### Scanning electron microscopy

The morphology of the HA and PHA was examined using a scanning electron microscope (SU8020, Hitachi, Japan).

#### X-ray photoelectron spectroscopy

X-ray photoelectron spectroscopic measurement (XPS, PHI OUANTERA-II SXM system, Japan/Uivac-PHI, INC) was used to obtain information about the surface element composition, chemical state, molecular structure and chemical bonds of the HA and PHA. An Al Kα ray was used as the excitation source, and the energy was 1486.6 eV. The XPS spectra were analyzed by the XPSPEAK software.

#### Solid-state ^13^C-nuclear magnetic resonance spectroscopy

To clarify the detailed distribution of the carbon functional groups of the HA and PHA, a solid-state ^13^C nuclear magnetic resonance spectrometer (Bruker AVANCE III HD 400 MHz, Switzerland) was used. The NMR measurement was carried out with the following parameters: H/X dual resonance solid probe, 4 mm ZrO_2_ rotor, speed of spinning: 5 kHz, ^13^C detection resonance frequency: 100.625 MHz, sampling time: 5.12 µs, spectral width: 100 kHz, 90° pulse-length: 4 µs, recycle delay time: 5.47 µs, number of scans: 4096 times, and chemical shift calibrated with standard glycine. Baseline correction and spectra integration were conducted using the MestReNova 9.0 software. The carbon types were divided into Alkyl carbons (C_Alk-H,R_, 0−45 ppm), methoxy and N-alkyl carbons (C_Alk-O,N_, 45−60 ppm), O-alkyl carbons (C_Alk-O_, 60−91 ppm), dioxide-alkyl carbon (C_Alk-di-O_, 91−110 ppm), aromatic carbon (C_Ar-H,R_, 110−142 ppm), O-aromatic carbon (C_Ar-O_, 142−156 ppm), carboxyl carbon (C_COO-H,R_, 156−186 ppm), and carbonyl carbon (C_C=O_, 186−230 ppm)^22,42^.

#### Gel permeation chromatography

The molecular weight distributions of the HA and PHA was determined by gel permeation chromatography (GPC, Shimazu LC-20A, Japan). The instrument configuration was as follows: LC20 high-performance liquid chromatography pump (Shimadzu, Japan), RID-20A refractive index detector (Shimadzu, Japan), TSKgel GMPWXL water phase gel chromatography column (TOSOH, Japan), Rheodyne 7725i manual six-port valve sampler (20 µl loop, USA), and HW-2000 GPC chromatography workstation. Polyethylene glycol samples were used as standard substances. HA or PHA (100 mg) was dissolved in 10 mL of a 0.1 M NaOH solution and then filtered through a 0.22 μm membrane filter before determination. The operation parameters were as follows: 35 °C column temperature, 0.1 N NaNO_3_ and 0.06% NaN_3_ aqueous solution as mobile phase, and 0.6 mL/min flow rate.

### Plant materials and incubation conditions

Hydroponic experiments were conducted in an environmentally controlled greenhouse (16/8 h light/dark cycle, 300 μmol m^−2^ s^−1^ light intensity, 28/21 °C temperature, 70% relative humidity) at the Dezhou experimental station, Chinese Academy of Agricultural Sciences, Shandong, China. Maize (*Zea mays* L. cv ZD 958) seeds were surface sterilized with a 0.1% NaClO solution for 10 min, washed 3 times with distilled water, and immersed in distilled water for 4 h. Then, the seeds were placed on moistened filter paper at 25 °C in the dark for 4 days until germination. After the endosperm was moved, the seedlings were transplanted to culture containers filled with Hoagland nutrient solution (5.0 mM Ca(NO_3_)_2_; 5.0 mM KNO_3_; 2.0 mM MgSO_4_; 1.0 mM KH_2_PO_4_; 45 μM HBO_3_; 10 μM MnCl_2_; 0.8 μM ZnSO_4_; 0.3 μM CuSO_4_; 0.4 μM Na_2_MoO_4_; 0.02 μM EDTA-Fe) with HA or PHA^39,43^. The added amounts of HA and PHA were 0, 10, 20, and 50 mg C/L, code named CK, HA10 or PHA10, HA20 or PHA20, HA50 or PHA50, respectively. Each treatment was repeated five times. The pH of the nutrient solutions was adjusted to 6.1, and they were renewed every 72 h. The entire hydroponic experiment lasted for 30 days, and then the plants were harvested.

### Sampling and laboratory analyses

The harvested plant was divided into roots, stalks and leaves, and each part was washed with distilled water, drained with blotting paper, and weighed to obtain the fresh weight. Subsequently, they were oven-dried at 105 °C for 30 min and at 65 °C to constant weight and weighed to obtain the dry weight. The dried samples were ground for N, P, and K analyses. After wet digestion with H_2_SO_4_–H_2_O_2_, the N, P, and K concentrations in the maize plant were determined by using a Kjeldahl apparatus (KDY-9820, Beijing Tongrunyuan Electromechanical Technology Co. Ltd., China), UV–vis spectrometer (UV-5500PC, Shanghai Precision Instruments Co., Ltd., China), and flame photometer (FP6400, Shanghai Jinpeng Analytical Instrument Co. Ltd., China), respectively^44^.

### Calculations and statistical analyses

The difference between the HA and the PHA in the elemental composition was compared by analysis of variance (ANOVA, SAS 9.1, SAS Institute Inc., NC, USA) with the least significant difference (LSD) test (α = 0.05). The differences between HA and PHA with different addition amount on plant growth stimulation were compared with the Tukey’s HSD test (α = 0.05). Graphs were compiled using the Origin 9.0 software.

## Supplementary information

Supplementary file1
