# Supplementary material for: Combining humic acid with phosphate fertilizer affects humic acid structure and its stimulating efficacy on the growth and nutrient uptake of maize seedlings
Source: Sci Rep. 2020 Oct 15;10:17502. doi: 10.1038/s41598-020-74349-6 (PMC7562911; doi:10.1038/s41598-020-74349-6)
Supplement: Supplementary file 1 — Supplementary file1 [file 41598_2020_74349_MOESM1_ESM.pdf]

Combining humic acid with phosphate fertilizer affects humic acid structure and its stimulating efficacy on the growth and nutrient uptake of maize seedlings

Jianyuan Jing,[a] Shuiqin Zhang,[a] Liang Yuan,[a] Yanting Li,[a] Zhian Lin,[a] Qizhong Xiong,[b] Bingqiang Zhao,[a*]

[a] *Key Laboratory of Plant Nutrition and Fertilizer, Ministry of Agriculture and Rural Affairs / Institute of Agricultural Resources and Regional Planning, Chinese Academy of Agricultural Sciences, Beijing 100081, China*

[b] *Anhui Province Key Laboratory of Farmland Ecological Conservation and Pollution Prevention, School of Resources and Environment, Anhui Agricultural University, Hefei 230036, China*

[*] Corresponding author.

Wavenumber (cm^{-1})	3500-3200	1707/1709	1602/1605	1421/1417	1227/1237	764/766	642
HA	23.12	10.45	14.67	12.42	27.59	6.21	5.55
PHA	26.09	12.39	16.33	12.69	26.83	3.40	2.28

Table S1. Relative absorption intensity of FT-IR spectra of HA and PHA.

Sample	Content of acidic functional groups (mmol/g)			Ratios of acidic functional groups of total acidity (%)	
	Total acidity	COOH	Ph-OH	COOH	Ph-OH
HA	3.16 ± 0.08	2.07 ± 0.10	1.09 ± 0.08	65.51	34.49
PHA	3.10 ± 0.02	2.08 ± 0.07	1.02 ± 0.08	67.10	32.90
HA7	1.23 ± 0.13	0.27 ± 0.01	0.96 ± 0.13	21.95	78.05
PHA7	1.21 ± 0.05	0.31 ± 0.04	0.09 ± 0.07	25.62	74.38

Table S2. Acid functional group content and distribution of HA and PHA.

C Type	HA		PHA	
	Binding energy (ev)	Relative amount (%)	Binding energy (ev)	Relative amount (%)
C-C	283.04	43.2	283.01	35.2
C-H	283.82	32.8	283.76	42.5
C-O	286.00	8.3	286.00	9.1
C=O	287.50	15.6	287.48	13.2

Table S3. XPS peaks (C1s) attribution and the relative content of HA and PHA.

N Type	HA		PHA	
	Binding energy (ev)	Relative amount (%)	Binding energy (ev)	Relative amount (%)
Pyridine N-6	398.27	88.8	398.37	91.6
Pyrrole N-5	399.50	11.2	399.49	8.4

Table S4. XPS peaks (N1s) attribution and the relative content of HA and PHA.

O Type	HA		PHA	
	Binding energy (ev)	Relative amount (%)	Binding energy (ev)	Relative amount (%)
-OH	530.11	43.5	530.20	43.6
-C-CO-O-	531.20	26.2	531.23	29.3
-C-O-C-	532.14	23.7	532.18	27.1
-CO-O-C-	533.6	6.6	-	-

Table S5. XPS peaks (O1s) attribution and the relative content of HA and PHA.

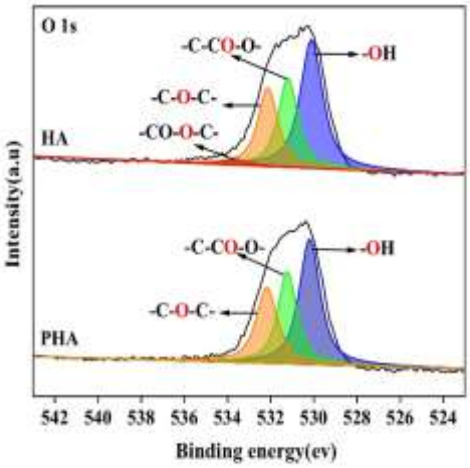

Figure S1. X-ray Photoelectron spectroscopy (O1s) of HA and PHA.

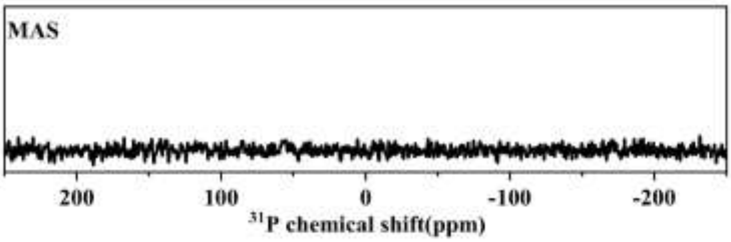

Figure S2. ^{31}P NMR analysis of PHA using MAS techniques.
